# Non‐Linear Threshold Effect of Dynamic Estimated Plasma Volume Status on Revealing In‐Hospital Mortality Risk in Sepsis

**DOI:** 10.1002/kjm2.70278

**Published:** 2026-08-26

**Authors:** Zhi‐An Si, Sheng‐Bing Xia, Yang Zou, Xiao‐Yan Wang

**Affiliations:** ^1^ Department of Oncology The 989th Hospital of the Joint Logistics Support Force of the PLA Luoyang China; ^2^ Department of Critical Care Medicine The 989th Hospital of the Joint Logistics Support Force of the PLA Luoyang China; ^3^ Department of Cardiology The 989th Hospital of the Joint Logistics Support Force of the PLA Luoyang China; ^4^ Department of Clinical Nutrition The First Affiliated Hospital of Chengdu Medical College Chengdu China

**Keywords:** estimated plasma volume status, in‐hospital mortality, risk stratification, sepsis, threshold effect

## Abstract

This retrospective cohort study evaluated the association between estimated plasma volume status (ePVS) and in‐hospital mortality in adult patients with sepsis from the MIMIC‐IV (v2.2) database. After excluding patients with early death, readmission, red blood cell transfusion, malignancy, or missing ePVS data, 17,403 patients meeting Sepsis‐3 criteria were included. ePVS was calculated using hemoglobin and hematocrit. Early ePVS (ePVS1), late‐ICU ePVS (ePVS2), and ePVS change were analyzed using multivariable logistic regression, restricted cubic splines, threshold analysis, prediction metrics, Kaplan–Meier analysis, subgroup analysis, and sensitivity analyses. In‐hospital mortality was 13.2%. ePVS2 and ePVS change were significantly higher in non‐survivors, whereas the difference in ePVS1 was small. After adjustment, ePVS2 (adjusted odds ratio [OR] = 1.14, 95% confidence interval [CI]: 1.11–1.18, *p* < 0.001) and ePVS change (adjusted OR = 1.04, 95% CI: 1.02–1.05, *p* < 0.001) remained independently associated with in‐hospital mortality, while ePVS1 was not significant. Non‐linear and threshold analyses suggested lower risk at ePVS2 of approximately 6–8 dL/g and ePVS change of −4.5 to 2, with increased risk at ePVS2 ≥ 8 dL/g or ePVS change ≥ 2. Late‐ICU ePVS2 and ePVS change may supplement volume‐status assessment and short‐term prognostic stratification in sepsis, but prospective validation is needed.

**Abbreviations**
AKI: acute kidney injury
AUC: area under the curve
BUN: blood urea nitrogen
CHF: congestive heart failure
CI: confidence interval
CPD: chronic pulmonary disease
Cr: creatinine
ePVS: estimated plasma volume status
HB: hemoglobin
Hct: hematocrit
ICU: intensive care unit
IDI: integrated discrimination improvement
MI: myocardial infarction
MIMIC‐IV: medical information mart for intensive care IV
NRI: net reclassification improvement
OR: odds ratio
PLT: platelet
RBC: red blood cell
RD: renal disease
ROC: receiver operating characteristic curve
RRT: renal replacement therapy
SAPS II: simplified acute physiology score II
SOFA: sequential organ failure assessment
WBC: white blood cell

## 1 Introduction

Sepsis is defined as life‐threatening organ dysfunction caused by a dysregulated host response to infection, representing one of the most common syndromes with a high mortality rate in critical care medicine [1, 2]. The Global Burden of Disease study indicated approximately 48.9 million incident cases of sepsis and 11.0 million sepsis‐related deaths worldwide in 2017, suggesting that it remains a major public health problem [3]. During sepsis development, abnormal volume status underlies key pathophysiological processes, including tissue hypoperfusion, microcirculatory dysfunction, and organ failure; therefore, fluid therapy remains a crucial foundation of hemodynamic support in sepsis [1, 4, 5]. However, fluid resuscitation has dual effects. Volume depletion can exacerbate perfusion disorders, whereas volume overload may lead to pulmonary edema, interstitial edema, and further deterioration of organ function [4, 6]. Consequently, accurately identifying volume status and implementing precise fluid management at different stages of sepsis are critical for improving patient prognosis.

Current clinical methods for evaluating volume status include central venous pressure, echocardiography, pulse pressure variation, stroke volume variation, and the passive leg raise test, all of which have limitations to varying degrees [4, 7]. Static indicators, such as central venous pressure, have a limited predictive ability for fluid responsiveness, while dynamic indicators are easily influenced by factors such as mechanical ventilation, spontaneous breathing, arrhythmias, and operator experience, limiting their clinical applicability [4, 8]. Although ultrasound provides extensive hemodynamic information, it requires high equipment standards and personnel training, making simple, continuous, and standardized high‐frequency monitoring difficult to achieve [4, 9]. Concurrently, an increasing number of studies suggest that positive fluid balance and fluid overload are closely associated with poorer outcomes in critically ill patients, indicating that fluid management in sepsis must focus not only on initial resuscitation but also on stabilization and de‐resuscitation during the later disease course [10, 11]. Therefore, there is an urgent clinical need for a quantifiable indicator of volume status that is derived from routine tests, easy to dynamically re‐evaluate, and highly accessible.

The estimated plasma volume status (ePVS), calculated based on hemoglobin (HB) and hematocrit (Hct), is characterized by its simplicity, low cost, and good reproducibility. Recently, it has gradually gained attention in studies of heart failure, acute respiratory distress syndrome, and critical care [12]. In sepsis, Kim et al. found that ePVS was correlated with fluid input volume and mortality risk, suggesting it could serve as a simple prognostic tool [13]. Furthermore, a 2024 study based on the Medical Information Mart for Intensive Care IV (MIMIC‐IV) database revealed a J‐shaped relationship between ePVS and 28‐day mortality risk in patients with septic shock, suggesting potential non‐linear relationships and threshold characteristics between ePVS and adverse outcomes [14]. These results suggest that ePVS may not only reflect volume load but also provide additional information for risk stratification and fluid management in sepsis [13, 14]. However, existing studies have mostly focused on ePVS at a single early time point, with insufficient attention to late‐ICU ePVS values and their dynamic changes during the disease course. Systematic evidence on whether stable non‐linear relationships and clear risk thresholds exist between ePVS at different disease stages and in‐hospital mortality remains lacking [13, 14].

Based on this, utilizing the MIMIC‐IV (v2.2) database, this study included adult patients with sepsis meeting the Sepsis‐3 criteria, primarily evaluating the relationship between early ePVS (ePVS_1_), the last ePVS during the intensive care unit (ICU) stay (ePVS_2_), their dynamic change indicator (ePVS change), and in‐hospital mortality. Simultaneously, this study further explored whether a non‐linear relationship and a threshold effect exist between these indicators and mortality risk, and evaluated their incremental predictive value for traditional risk models. From the perspective of dynamic volume assessment, this study aimed to provide complementary evidence for understanding stage‐specific ePVS patterns, short‐term prognostic stratification, and clinically recognizable risk intervals in patients with sepsis, while avoiding interpretation of late‐ICU ePVS_2_ as a strict baseline predictor.

## 2 Materials and Methods

### 2.1 Database

This was a retrospective cohort study. The research data were extracted from the MIMIC‐IV (v2.2) database established by Johnson et al. [15]. The patients in the database were admitted to the ICU at Beth Israel Deaconess Medical Center in the United States between 2008 and 2019. The study was reported in accordance with the Reporting of Studies Conducted using Observational Routinely‐Collected Health Data (RECORD) statement, an extension of STROBE for studies using routinely collected health data. To obtain database access, we completed the online training course provided by the National Institutes of Health and passed the test on protecting human research subjects. One author (Xiaoyan Wang) obtained access to the database and was responsible for data extraction (Record ID: 60201641).

### 2.2 Study Population

The inclusion criteria were adult patients in the MIMIC‐IV (v2.2) database who met the diagnostic criteria for sepsis. The diagnosis of sepsis was based on the Sepsis‐3 criteria [1]. The exclusion criteria were as follows: (1) age < 18 years; (2) hospital or ICU readmissions; (3) length of ICU stay < 24 h or death within 24 h after ICU admission; (4) history of red blood cell (RBC) transfusion within 48 h after the diagnosis of sepsis or during the ICU stay; (5) concomitant malignant tumors, including lymphoma and leukemia (excluding skin malignancies); and (6) missing ePVS data. Patients who received RBC transfusion were excluded because transfusion directly changes hemoglobin and hematocrit, which are the mathematical inputs used to calculate ePVS, and could therefore introduce measurement distortion in ePVS‐based analyses.

### 2.3 Calculation Formula for ePVS and Indicator Definitions

ePVS was calculated using the Duarte formula [16]. ePVS change was calculated using the Strauss formula [17] to indicate the relative change in plasma volume status between two specific time points, as detailed below:

$$\text{ePVS}\;\left(\mathrm{dL}/g\right)=\frac{100-\text{Hematocrit}\;\left(\%\right)}{\text{Hemoglobin}\;\left(g/\mathrm{dL}\right)}$$

$$\begin{array}{cc} {\text{ePVS}}_{\text{change}}\left(\%\right)= & \;100\times \left[\frac{\text{Hemoglobin}\;1\;\left(g/\mathrm{dL}\right)}{\text{Hemoglobin}\;2\;\left(g/\mathrm{dL}\right)}\times \left(\frac{1-\text{Hematocrit}\;2\;\left(\%\right)}{1-\text{Hematocrit}\;1\;\left(\%\right)}\right)\right]\\ & \;-100 \end{array}$$

The following parameters were established according to distinct time points: ePVS_1_ is the initial ePVS measurement obtained within the period from 24 h before to 4 h after sepsis diagnosis; ePVS_2_ is the defined as the final ePVS value documented during the ICU stay, representing the plasma volume status at the later stage of the disease course; consequently, the analysis of ePVS_2_ primarily evaluated the correlation between mid‐to‐late stage volume status and outcomes, rather than serving as a strict baseline predictive effect; ePVS change is the parameter representing the variation between ePVS_2_ and ePVS_1_.

Among these, ePVS_1_ primarily reflects the plasma volume status in the early stage of sepsis diagnosis, ePVS_2_ reflects the last recorded plasma volume status during the ICU stay, and ePVS change reflects the dynamic changes in plasma volume status during hospitalization.

### 2.4 Variable Extraction

Structured Query Language was used to extract clinical variables before and after sepsis diagnosis, including demographic characteristics, disease severity, comorbidities, interventions, vital signs, and laboratory indicators.

The extracted demographic characteristics included age, sex, weight, race, and ICU type. Disease severity was evaluated using Simplified Acute Physiology Score II (SAPS II) and Sequential Organ Failure Assessment (SOFA) score. Interventions included whether mechanical ventilation was received within 24 h before to 24 h after the diagnosis of sepsis, and whether renal replacement therapy (RRT) was received; meanwhile, the use of vasoactive drugs was recorded, and the norepinephrine equivalent was calculated. Vital signs included heart rate, systolic blood pressure, diastolic blood pressure, respiratory rate, and peripheral oxygen saturation. Laboratory indicators included RBC, white blood cell (WBC), platelet (PLT), blood urea nitrogen (BUN), creatinine (Cr), serum sodium, serum potassium, serum chloride, anion gap, bicarbonate, blood glucose, pH, partial pressure of oxygen, and partial pressure of carbon dioxide.

For variables recorded multiple times before and after the diagnosis of sepsis, the value rules were as follows: (1) vital signs: the value reflecting the most severe condition within 6 h before to 6 h after the diagnosis of sepsis was selected; (2) acute kidney injury (AKI) staging: the highest stage within 6 h before to 6 h after the diagnosis of sepsis was selected; (3) laboratory indicators: the first test value within 24 h before to 4 h after the diagnosis of sepsis was selected; (4) mechanical ventilation: whether mechanical ventilation was received within 24 h before to 24 h after the diagnosis of sepsis was recorded; and (5) RRT: whether RRT was received within 24 h before to 24 h after the diagnosis of sepsis was recorded.

We further extracted the occurrence of septic shock and AKI, along with AKI staging. AKI [18] and its stages were defined according to the Kidney Disease Improving Global Outcomes criteria. Septic shock [1] was defined as patients requiring vasoactive drugs within 24 h after sepsis diagnosis, including norepinephrine, epinephrine, phenylephrine, vasopressin, dopamine, metaraminol, and angiotensin II [19]. Comorbidities were identified based on ICD‐9/ICD‐10 diagnostic codes recorded in the MIMIC‐IV (v2.2) database, including congestive heart failure (CHF), myocardial infarction (MI), chronic pulmonary disease (CPD), and renal disease (RD) (Table S1). Additionally, the Charlson comorbidity index was recorded.

### 2.5 Outcome Indicators

The primary outcome of this study was in‐hospital mortality. Secondary outcomes included 28‐day and 90‐day mortality, which were utilized for survival analysis.

### 2.6 Statistical Analysis

Continuous variables were expressed as mean (standard deviation) or median (interquartile range), depending on their distribution, while categorical variables were presented as counts and percentages. Comparisons of continuous variables between groups were conducted using Student's *t*‐test or the Mann–Whitney *U* test, and comparisons of categorical variables were performed using the Chi‐square test or Fisher's exact test.

The primary outcome of this study was in‐hospital mortality. The main exposure variables were ePVS_1_, ePVS_2_, and ePVS change. Based on the results of the threshold analysis, ePVS_1_ and ePVS_2_ were further categorized into three groups (minimum to < 6, 6 to < 8, and 8 dL/g to maximum), and ePVS change was categorized into three groups (< −4.5, −4.5 to < 2, and ≥ 2) for categorical analysis.

First, a binomial logistic regression model was used to evaluate the association between ePVS indicators and in‐hospital mortality. Potential confounding factors with clinical significance were included a priori in the multivariable model, including age, sex, Charlson comorbidity index, SOFA, SAPS II, septic shock, AKI, mechanical ventilation, and RRT. The regression results were expressed as odds ratios (ORs) and their 95% confidence intervals (CIs). The primary multivariable models used predefined representative ePVS measures, including early ePVS_1_, the last ePVS during the ICU stay, and ePVS change, rather than a full time‐updated or longitudinal modeling framework. This approach was chosen to maintain interpretability and because complete timestamped longitudinal trajectories of all relevant covariates were not available in the cleaned analysis matrix.

To explore the non‐linear relationship between ePVS indicators and in‐hospital mortality, restricted cubic spline models were fitted with predefined knots placed at empirical percentiles of each ePVS indicator. Models with 3, 4, and 5 knots were compared, and the final specification was selected based on model fit, parsimony, and curve stability. The final curves were used to identify visually and statistically supported low‐risk intervals. Non‐linearity was assessed by likelihood‐ratio tests comparing spline models with corresponding linear models. Subsequently, a piecewise linear regression model was constructed to evaluate the threshold effect of ePVS_1_, ePVS_2_, and ePVS change, and the relatively low‐risk interval was determined accordingly. Non‐linear curves were expressed as ORs and their 95% CIs, and centered based on the reference value.

To evaluate the incremental predictive value of ePVS indicators, a pre‐specified basic model was constructed, including age, sex, Charlson comorbidity index, SOFA, and SAPS II, and ePVS_2_ or ePVS change was added to form an extended model. A 5‐fold stratified cross‐validation was used to obtain out‐of‐fold predicted probabilities, and receiver operating characteristic (ROC) curves and areas under the curve (AUCs) were calculated accordingly. Calibration curves were drawn using decile grouping of predicted probabilities to evaluate consistency between the model's predicted and observed probabilities. Bootstrap resampling was then performed 400 times to calculate AUC differences, net reclassification improvement (NRI), integrated discrimination improvement (IDI), and their 95% CIs, thereby assessing the incremental predictive performance of ePVS indicators relative to the basic model.

The Kaplan–Meier method was used to estimate 28‐day and 90‐day survival curves for patients in different ePVS_2_ groups, and the log‐rank test was used to compare survival differences between groups. Because follow‐up was indexed from ICU admission whereas ePVS_2_ was defined using the final ePVS value during the ICU stay, these analyses were considered supportive descriptive analyses rather than strict baseline prediction analyses.

Subgroup analyses stratified by the presence of septic shock were conducted to assess the consistency of the association between ePVS_1_, ePVS_2_, ePVS change, and in‐hospital mortality. Regression models were constructed for continuous and categorical variables, and interaction terms were included to examine differences in effects across subgroups. The results of the subgroup analyses were presented using forest plots.

Missing data are detailed in the Supporting Information (Table S1). The regression analyses used available cases with complete data for the variables required in each model. For prediction model construction, median imputation for continuous variables and mode imputation for binary variables were performed within each cross‐validation training fold to mitigate information leakage. To verify the robustness of the results, sensitivity analyses were subsequently performed, including the exclusion of extreme ePVS_2_ values (outside the 1st to 99th percentiles), the exclusion of patients receiving RRT, and the exclusion of patients with severe anemia (HB1 < 7 g/dL or HB2 < 7 g/dL), followed by the reconstruction of multivariable logistic regression models.

All statistical analyses were conducted using Python, primarily using libraries such as pandas, numpy, scipy, statsmodels, scikit‐learn, matplotlib, and patsy. All tests were two‐sided, and a *p*‐value < 0.05 was considered statistically significant.

### 2.7 Ethical Approval and Informed Consent

Not applicable. This study adhered to the Reporting of Studies Conducted using Observational Routinely‐Collected Health Data (RECORD) statement.

## 3 Results

### 3.1 Formation of a Stable Analysis Cohort After Screening of Study Subjects

**FIGURE 1:**
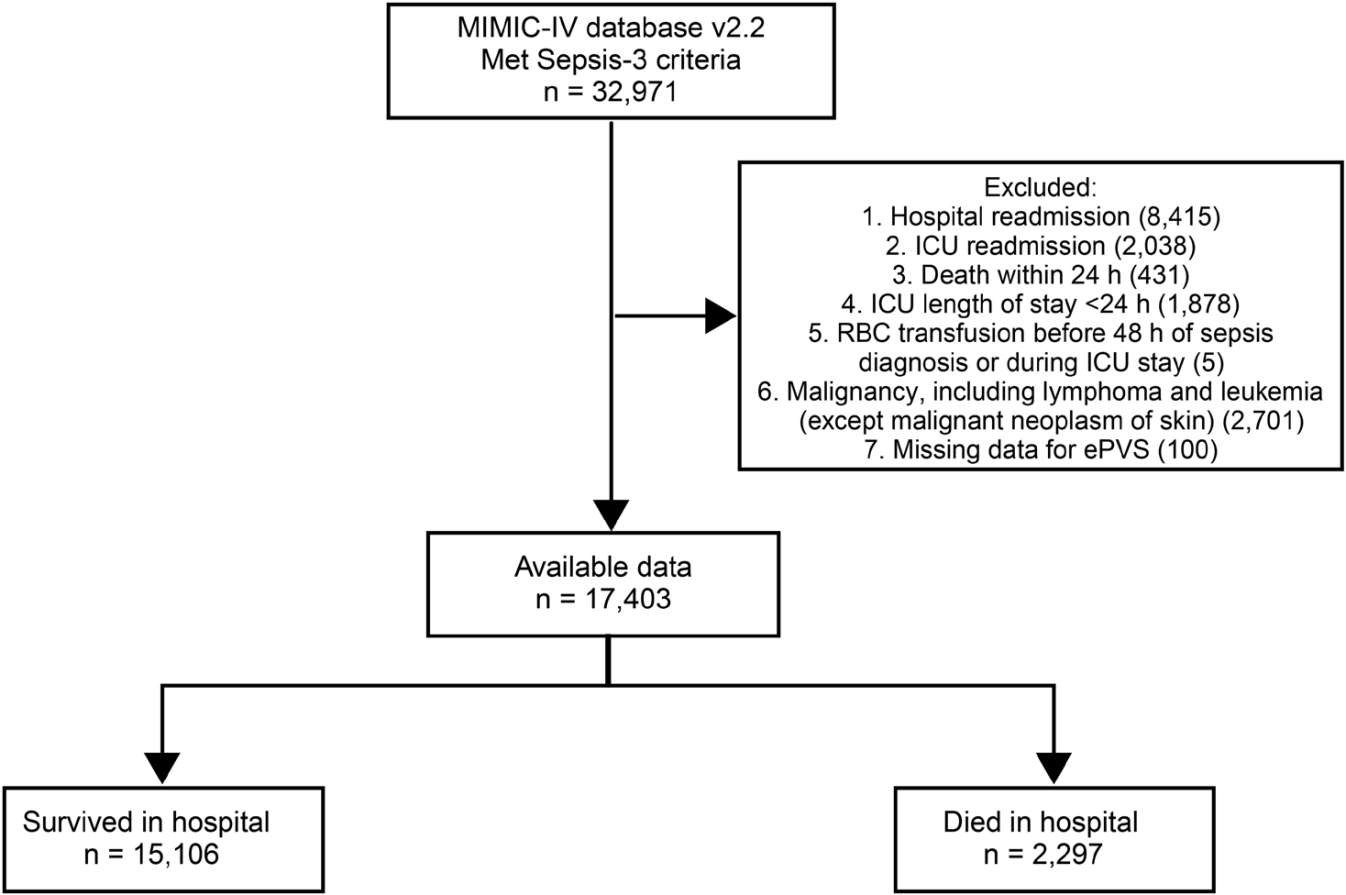
Flowchart of study population selection. Based on the MIMIC‐IV (v2.2) database, sepsis patients were screened according to the Sepsis‐3 criteria. After sequentially excluding patients with hospital readmission, ICU readmission, death within 24 h, ICU stay less than 24 h, RBC transfusion within 48 h after sepsis diagnosis or during the ICU stay, malignant tumors, and missing ePVS data, 17,403 patients were finally included, of which 15,106 survived in the hospital, and 2297 died in the hospital.

According to the Sepsis‐3 criteria, a total of 32,971 patients with sepsis were screened from the MIMIC‐IV (v2.2) database. After excluding patients with hospital readmission, ICU readmission, death within 24 h, ICU stay less than 24 h, RBC transfusion within 48 h after sepsis diagnosis and during the ICU stay, concurrent malignancies, and missing ePVS data, 17,403 patients were finally included in the analysis. Among them, 15,106 survived in‐hospital, and 2297 died in‐hospital, with an in‐hospital mortality rate of 13.2% (Figure 1).

### 3.2 Deceased Patients Exhibited Greater Disease Severity and More Pronounced Plasma Volume Abnormalities

A comparison of baseline characteristics is presented in Table 1. Compared with the survival group, patients in the death group were older (70.3 ± 15.9 vs. 65.4 ± 16.6 years), had higher SOFA and SAPS II scores, and demonstrated a significantly elevated Charlson comorbidity index. The death group had higher proportions of CHF, MI, CPD, and RD, while septic shock and AKI were also more common, especially with a significantly increased proportion of AKI stage 3. Regarding treatment, the proportions of patients receiving RRT within 24 h and 7 days, as well as mechanical ventilation within 24 h and 48 h, were significantly higher in the death group than in the survival group.

**TABLE 1:** Baseline characteristics of the study population according to in‐hospital survival status.

Variables	Total (*n* = 17,403)	Survivor (*n* = 15,106)	Non‐survivor (*n* = 2297)	*p*
Age (years)	66.0 ± 16.6	65.4 ± 16.6	70.3 ± 15.9	< 0.001
Sex				
Male	10,048 (57.7)	8805 (58.3)	1243 (54.1)	< 0.001
Race				
White	11,588 (66.6)	10,256 (67.9)	1332 (58)	< 0.001
Black	1329 (7.6)	1179 (7.8)	150 (6.5)	
Other	1764 (10.1)	1584 (10.5)	180 (7.8)	
Unknown	2722 (15.6)	2087 (13.8)	635 (27.6)	
Weight (kg)	83.4 ± 22.8	83.8 ± 22.6	80.9 ± 23.5	< 0.001
Care unit				
CVICU/CCU	6028 (34.6)	5538 (36.7)	490 (21.3)	< 0.001
MICU/GICU	6594 (37.9)	5511 (36.5)	1083 (47.1)	
Neuro/T/SICU	4781 (27.5)	4057 (26.9)	724 (31.5)	
Severity of illness				
SOFA score	3.6 ± 1.9	3.5 ± 1.8	4.3 ± 2.5	< 0.001
SAPSII score	36.6 ± 13.6	34.9 ± 12.4	48.2 ± 15.3	< 0.001
Comorbidities				
Charlson comorbidity index	4.0 (3.0, 6.0)	4.0 (2.0, 6.0)	6.0 (4.0, 8.0)	< 0.001
Congestive heart failure				
No	12,243 (70.3)	10,818 (71.6)	1425 (62)	< 0.001
Yes	5160 (29.7)	4288 (28.4)	872 (38)	
Myocardial Infarction				
No	14,283 (82.1)	12,510 (82.8)	1773 (77.2)	< 0.001
Yes	3120 (17.9)	2596 (17.2)	524 (22.8)	
Chronic pulmonary disease				
No	12,898 (74.1)	11,256 (74.5)	1642 (71.5)	0.002
Yes	4505 (25.9)	3850 (25.5)	655 (28.5)	
Renal disease				
No	13,745 (79.0)	12,078 (80)	1667 (72.6)	< 0.001
Yes	3658 (21.0)	3028 (20)	630 (27.4)	
Norepinephrine equivalent	0.0 (0.0, 0.5)	0.0 (0.0, 0.4)	0.2 (0.0, 3.5)	< 0.001
Septic shock				
No	9819 (56.4)	8815 (58.4)	1004 (43.7)	< 0.001
Yes	7584 (43.6)	6291 (41.6)	1293 (56.3)	
AKI stage				
No AKI	6737 (38.7)	6220 (41.2)	517 (22.5)	< 0.001
AKI Stage 1	3138 (18.0)	2790 (18.5)	348 (15.2)	
AKI Stage 2	5264 (30.2)	4555 (30.2)	709 (30.9)	
AKI Stage 3	2264 (13.0)	1541 (10.2)	723 (31.5)	
Interventions, *n* (%)				
RRT within 24 h				
No	17,095 (98.2)	14,942 (98.9)	2153 (93.7)	< 0.001
Yes	308 (1.8)	164 (1.1)	144 (6.3)	
RRT within 7 days				
No	16,661 (95.7)	14,725 (97.5)	1936 (84.3)	< 0.001
Yes	742 (4.3)	381 (2.5)	361 (15.7)	
MV within 24 h				
No	8902 (51.2)	8022 (53.1)	880 (38.3)	< 0.001
Yes	8501 (48.8)	7084 (46.9)	1417 (61.7)	
MV within 48 h				
No	8509 (48.9)	7755 (51.3)	754 (32.8)	< 0.001
Yes	8894 (51.1)	7351 (48.7)	1543 (67.2)	
Vital signs				
Heart Rate (bpm)	99.5 ± 20.4	98.9 ± 19.9	103.6 ± 22.8	< 0.001
Respiratory Rate (bpm)	25.7 ± 6.4	25.5 ± 6.3	27.4 ± 6.5	< 0.001
SBP (mmHg)	94.6 ± 17.7	94.9 ± 17.3	92.6 ± 19.9	< 0.001
DBP (mmHg)	48.8 ± 11.4	49.0 ± 11.1	47.2 ± 12.7	< 0.001
Pulse Oxygen Saturation (%)	93.7 ± 5.3	93.9 ± 4.9	92.3 ± 7.1	< 0.001
Estimated Plasma Volume (ePVS)				
ePVS1 (dL/g)	6.6 ± 2.2	6.6 ± 2.2	6.7 ± 2.3	0.012
ePVS2 (dL/g)	7.3 ± 1.8	7.2 ± 1.7	7.9 ± 2.3	< 0.001
ePVSchange	0.0 ± 3.7	‐0.1 ± 3.6	0.2 ± 4.8	< 0.001
Laboratory variables				
Hemoglobin1 (g/dL)	10.9 ± 2.3	10.9 ± 2.2	10.7 ± 2.4	0.011
Hematocrit1 (%)	32.9 ± 6.7	32.9 ± 6.6	33.1 ± 7.2	0.189
Hemoglobin2 (g/dL)	9.9 ± 1.7	10.0 ± 1.7	9.4 ± 1.9	< 0.001
Hematocrit2 (%)	30.2 ± 5.1	30.3 ± 4.9	29.2 ± 5.7	< 0.001
Red Blood Cells (m/μL)	3.8 ± 0.8	3.8 ± 0.8	3.7 ± 0.9	< 0.001
White Blood Cells (K/μL)	11.2 (7.9, 15.8)	11.0 (7.8, 15.5)	12.5 (8.7, 17.5)	< 0.001
Platelet Count (K/μL)	196.0 (141.0, 265.0)	196.0 (143.0, 264.0)	194.0 (129.0, 270.0)	0.016
Urea Nitrogen (mg/dL)	21.0 (14.0, 34.0)	20.0 (14.0, 32.0)	30.0 (18.0, 47.0)	< 0.001
Creatinine (mg/dL)	1.0 (0.8, 1.6)	1.0 (0.8, 1.5)	1.4 (0.9, 2.2)	< 0.001
Sodium (mEq/L)	138.2 ± 5.8	138.3 ± 5.6	138.0 ± 7.0	0.035
Potassium (mEq/L)	4.3 ± 0.9	4.3 ± 0.9	4.5 ± 1.0	< 0.001
Chloride (mEq/L)	103.0 ± 7.3	103.2 ± 7.2	101.6 ± 7.9	< 0.001
Anion Gap (mEq/L)	15.8 ± 5.1	15.5 ± 4.9	17.9 ± 5.8	< 0.001
Bicarbonate (mEq/L)	23.2 ± 5.0	23.4 ± 4.8	21.7 ± 5.9	< 0.001
Glucose (mg/dL)	158.0 (125.0, 197.0)	158.0 (125.0, 195.0)	160.0 (121.0, 214.0)	0.012
pH	7.4 ± 0.1	7.4 ± 0.1	7.3 ± 0.1	< 0.001
PaO_2_ (mmHg)	199.0 (98.0, 362.0)	221.0 (103.0, 374.0)	117.0 (79.0, 226.5)	< 0.001
PaCO_2_ (mmHg)	41.0 (36.0, 47.0)	41.0 (36.0, 46.0)	40.0 (34.0, 49.0)	0.004

*Note:* Continuous variables are presented as mean ± SD or median (IQR), and categorical variables as *n* (%). *p*‐values compare survivors and non‐survivors.

Abbreviations: AKI, acute kidney injury; ePVS, estimated plasma volume status; ICU, intensive care unit; MV, mechanical ventilation; RRT, renal replacement therapy; SAPS II, Simplified Acute Physiology Score II; SOFA, Sequential Organ Failure Assessment.

In terms of primary exposure indicators, ePVS_2_ was significantly higher in the death group than in the survival group (7.9 ± 2.3 vs. 7.2 ± 1.7 dL/g), and ePVS change was also greater (0.2 ± 4.8 vs. −0.1 ± 3.6). Although there was a statistical difference in ePVS_1_ between the two groups (6.7 ± 2.3 vs. 6.6 ± 2.2 dL/g), the magnitude of the difference was relatively small. Simultaneously, HB2 and Hct2 levels were lower in the death group, while WBC count, BUN, Cr, and anion gap were higher, indicating greater disease severity and more pronounced internal environmental disorders (Table 1). Associations between baseline characteristics and ePVS indices are summarized in Table S4, providing additional context for confounding assessment and covariate selection.

### 3.3 ePVS Showed a Significant Non‐Linear Relationship With In‐Hospital Mortality

**FIGURE 2:**
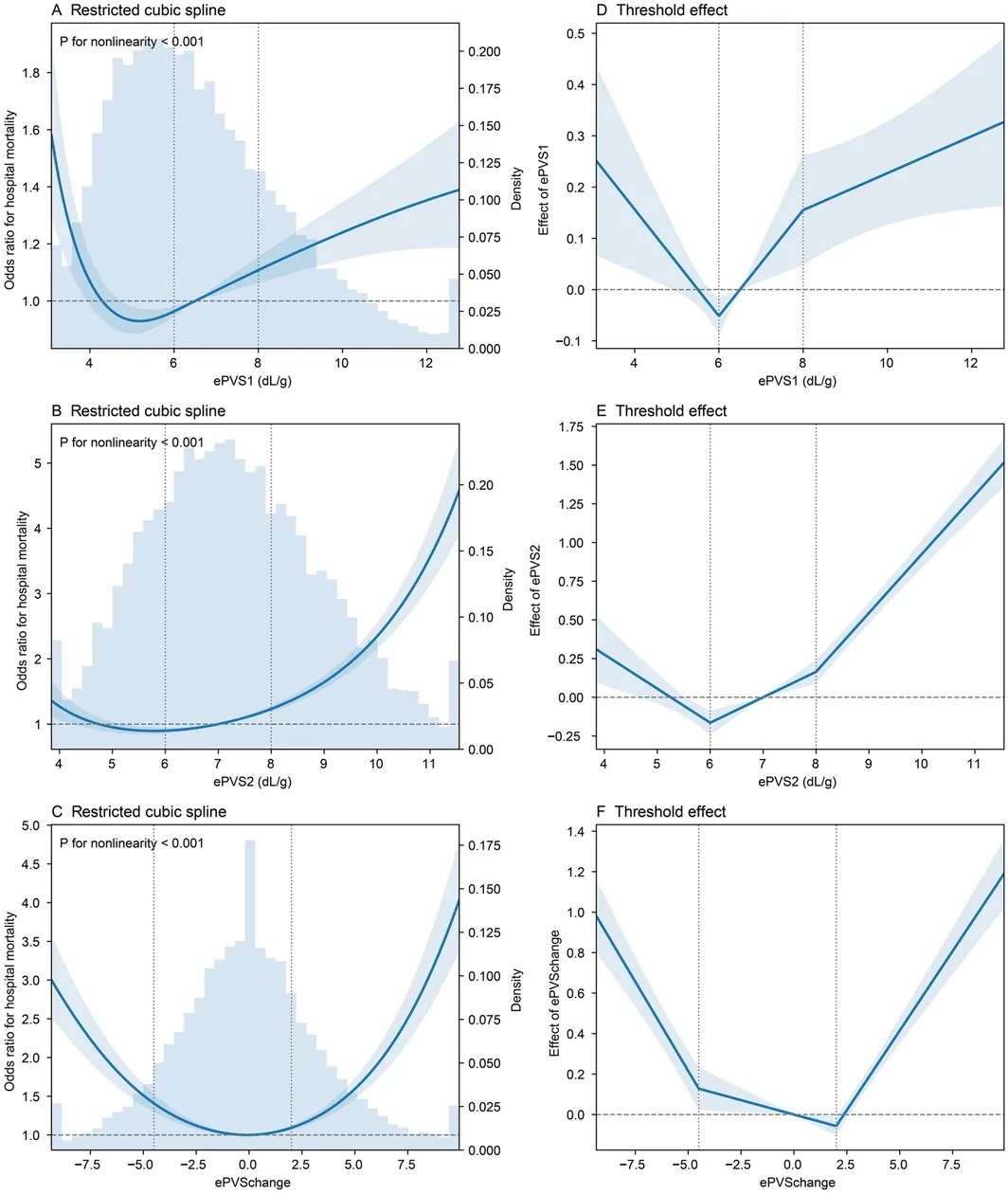
Restricted cubic spline analysis and threshold effect models of the association between ePVS and in‐hospital mortality. (A) Restricted cubic spline curve of the association between ePVS_1_ and in‐hospital mortality; (B) Restricted cubic spline curve of the association between ePVS_2_ and in‐hospital mortality; (C) Restricted cubic spline curve of the association between ePVS change and in‐hospital mortality; (D) Piecewise linear regression threshold effect plot for ePVS_1_; (E) Piecewise linear regression threshold effect plot for ePVS_2_; (F) Piecewise linear regression threshold effect plot for ePVS change. A total of 17,403 patients were included in the analysis. Both the restricted cubic spline models and piecewise linear regression models were adjusted for confounding factors, including age, sex, Charlson comorbidity index, SOFA score, SAPS II score, septic shock, AKI, mechanical ventilation, and RRT. In A–C, the solid lines represent the estimated effects, the shaded areas represent the 95% CIs, the background histograms represent the distribution density of the corresponding indicators, and the horizontal dashed lines indicate OR = 1; In D–F, the solid lines represent the fitted effects of the piecewise linear regression, the shaded areas represent the 95% CIs, the vertical dashed lines indicate the threshold cut‐off points, and the horizontal dashed lines represent the effect reference lines. ePVS stands for estimated plasma volume status. All tests were two‐sided, and *p <* 0.05 was considered statistically significant.

Restricted cubic spline analysis indicated that after adjusting for confounding factors, ePVS_1_, ePVS_2_, and ePVS change exhibited significant non‐linear relationships with in‐hospital mortality, with all *p*‐values for the non‐linear tests being < 0.001 (Figure 2). ePVS_1_ showed a non‐linear pattern with a weak independent association after full adjustment, whereas ePVS_2_ and ePVS change displayed U‐shaped relationships, indicating that excessively low or high levels were associated with higher mortality risks.

Based on the shape of the curves, ePVS_1_ and ePVS_2_ corresponded to lower mortality risks within the range of approximately 6–8 dL/g, while ePVS change was associated with lower risks within the interval of −4.5 to 2. When the parameters deviated from these ranges, mortality risk increased progressively. These findings suggest that the relationship between ePVS and in‐hospital mortality is not simply linear, but rather characterized by relatively low‐risk intervals (Figure 2).

### 3.4 Threshold Analysis Further Supported the Piecewise Risk Pattern of ePVS

**FIGURE 3:**
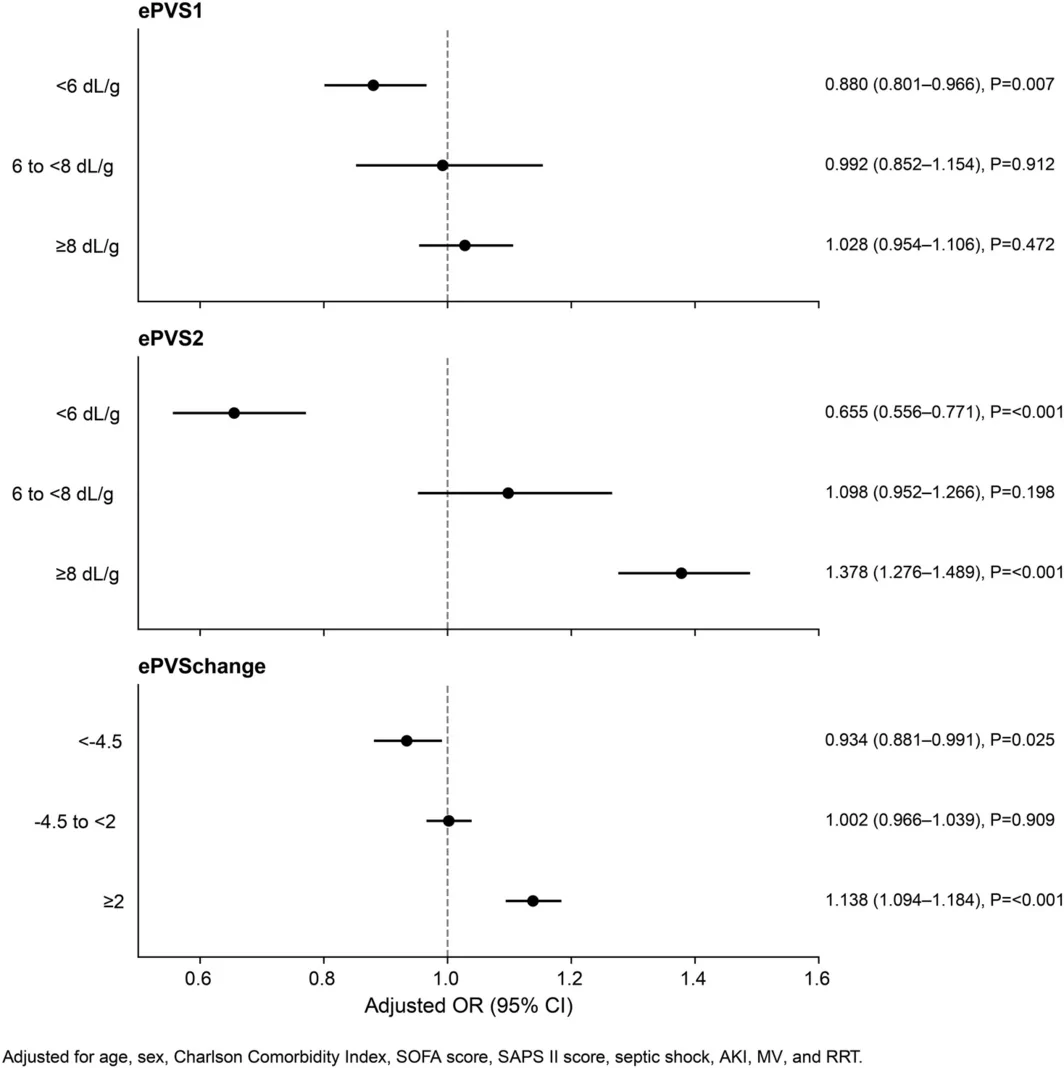
Piecewise linear regression results of threshold effects for ePVS indices.

Piecewise linear regression demonstrated that for ePVS_1_ from the minimum to < 6 dL/g, the OR was 0.880 (95% CI: 0.801–0.966, *p* = 0.007); no significant association was observed in the 6 to < 8 dL/g interval; and no significant risk increase was seen from 8 dL/g to the maximum. In contrast, the piecewise effect of ePVS_2_ was more pronounced: for ePVS_2_ from the minimum to < 6 dL/g, the OR was 0.655 (95% CI: 0.556–0.771, *p* < 0.001); no significant association was found in the 6 to < 8 dL/g interval; however, the mortality risk significantly increased from 8 dL/g to the maximum, with an OR of 1.378 (95% CI: 1.276–1.489, *p* < 0.001). ePVS change also demonstrated a clear threshold effect: for values below −4.5, the OR was 0.934 (95% CI: 0.881–0.991, *p* = 0.025); the association was not significant in the −4.5 to < 2 interval; and for values ≥ 2, the OR rose to 1.138 (95% CI: 1.094–1.184, *p* < 0.001). The likelihood ratio tests for all three parameters indicated that the piecewise models were statistically significant, with ePVS_2_ and ePVS change providing the strongest evidence (Table 2, Figure 3).

**TABLE 2:** Threshold effect analyses of ePVS indicators for in‐hospital mortality.

Model segment	Adjusted OR (95% CI)	*p*
Three‐piecewise linear regression model for ePVS1 (dL/g)		
ePVS1 < 6	0.880 (0.801–0.966)	0.007
ePVS1 ≥ 6 and < 8	0.992 (0.852–1.154)	0.912
ePVS1 ≥ 8	1.028 (0.954–1.106)	0.472
Likelihood ratio test		0.033
Three‐piecewise linear regression model for ePVS2 (dL/g)		
ePVS2 < 6	0.655 (0.556–0.771)	< 0.001
ePVS2 ≥ 6 and < 8	1.098 (0.952–1.266)	0.198
ePVS2 ≥ 8	1.378 (1.276–1.489)	< 0.001
Likelihood ratio test		< 0.001
Three‐piecewise linear regression model for ePVSchange		
ePVSchange < −4.5	0.934 (0.881–0.991)	0.025
ePVSchange ≥ −4.5 and < 2	1.002 (0.966–1.039)	0.909
ePVSchange ≥ 2	1.138 (1.094–1.184)	< 0.001
Likelihood ratio test		< 0.001

*Note:* Threshold effects were estimated using piecewise linear regression models. The ORs in the piecewise model represent segment‐specific changes along the continuous ePVS scale and should not be interpreted as categorical group comparisons. Odds ratios are adjusted for age, sex, Charlson Comorbidity Index, SOFA score, SAPS II score, septic shock, AKI, MV, and RRT.

Abbreviations: CI, confidence interval; ePVS, estimated plasma volume status.

These results suggest a clearer threshold‐dependent relationship between ePVS_2_ and ePVS change and mortality risk, with the risk increasing most significantly when ePVS_2_ was 8 dL/g to the maximum and ePVS change was ≥ 2.

### 3.5 ePVS_2_ and ePVS Change During the ICU Stay Are Independently Associated With In‐Hospital Mortality

After including ePVS as a continuous variable in the model and adjusting for age, sex, Charlson comorbidity index, SOFA score, SAPS II score, septic shock, AKI, mechanical ventilation, and RRT, ePVS_2_ remained independently and positively correlated with in‐hospital mortality, with an adjusted OR of 1.14 (95% CI: 1.11–1.18, *p <* 0.001). Similarly, ePVS change was significantly associated with in‐hospital mortality, with an adjusted OR of 1.04 (95% CI: 1.02–1.05, *p <* 0.001). However, ePVS_1_ showed no statistical significance after full adjustment, with an adjusted OR of 0.98 (95% CI: 0.96–1.00, *p =* 0.082).

**FIGURE 4:**
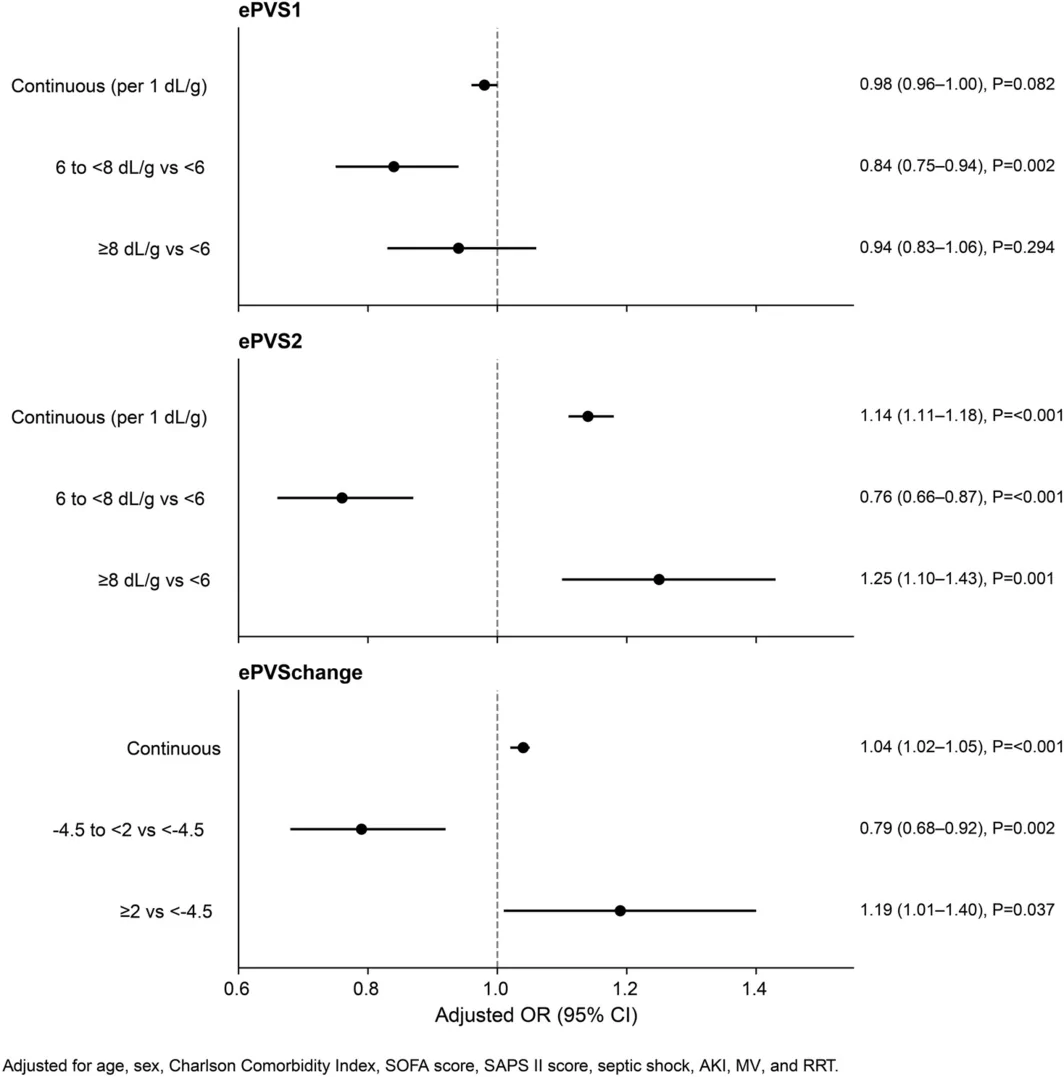
Multivariable logistic regression results of ePVS indicators and in‐hospital mortality.

Categorical analysis further indicated that, compared to the ePVS_2_ group from the minimum to < 6 dL/g, the ePVS_2_ 6 to < 8 dL/g group had a lower mortality risk, with an adjusted OR of 0.76 (95% CI: 0.66–0.87, *p* < 0.001); whereas the ePVS_2_ group from 8 dL/g to the maximum exhibited a significantly increased mortality risk, with an adjusted OR of 1.25 (95% CI: 1.10–1.43, *p* = 0.001). Regarding ePVS change, compared to the < −4.5 group, the −4.5 to < 2 group demonstrated a lower risk, with an adjusted OR of 0.79 (95% CI: 0.68–0.92, *p* = 0.002), while the ≥ 2 group showed an increased risk, with an adjusted OR of 1.19 (95% CI: 1.01–1.40, *p* = 0.037). ePVS_1_ only exhibited a lower risk in the 6 to < 8 dL/g group, while the group from 8 dL/g to the maximum was no longer significant after adjustment (Table 3, Figure 4).

**TABLE 3:** Associations of ePVS indicators with in‐hospital mortality.

Estimated plasma volume (ePVS)	Observations, *n* (%)	In‐hospital mortality, *n* (%)	Crude OR (95% CI)	Crude *p*	Adjusted OR (95% CI)	Adjusted *p*
ePVS1 (dL/g)						
Continuous	17,403	2297 (13.2)	1.02 (1.01–1.04)	0.012	0.98 (0.96–1.00)	0.082
< 6	7731	1000 (12.9)	1 (Ref)		1 (Ref)	
≥ 6 and < 8	5695	696 (12.2)	0.94 (0.85–1.04)	0.219	0.84 (0.75–0.94)	0.002
≥ 8	3977	601 (15.1)	1.20 (1.07–1.34)	0.001	0.94 (0.83–1.06)	0.294
*p* for trend	17,403			0.006		0.149
ePVS2 (dL/g)						
Continuous	17,403	2297 (13.2)	1.24 (1.21–1.27)	< 0.001	1.14 (1.11–1.18)	< 0.001
< 6	4108	436 (10.6)	1 (Ref)		1 (Ref)	
≥ 6 and < 8	7524	788 (10.5)	0.99 (0.87–1.11)	0.814	0.76 (0.66–0.87)	< 0.001
≥ 8	5771	1073 (18.6)	1.92 (1.71–2.17)	< 0.001	1.25 (1.10–1.43)	0.001
*p* for trend	17,403			< 0.001		< 0.001
ePVSchange						
Continuous	17,403	2297 (13.2)	1.02 (1.01–1.03)	0.001	1.04 (1.02–1.05)	< 0.001
< −4.5	1678	318 (19.0)	1 (Ref)		1 (Ref)	
≥ −4.5 and < 2	11,335	1248 (11.0)	0.53 (0.46–0.61)	< 0.001	0.79 (0.68–0.92)	0.002
≥ 2	4390	731 (16.7)	0.85 (0.74–0.99)	0.034	1.19 (1.01–1.40)	0.037
*p* for trend	17,403			0.030		< 0.001

*Note:* Adjusted models were controlled for age, sex, Charlson Comorbidity Index, SOFA score, SAPS II score, septic shock, AKI, MV, and RRT.

Abbreviations: CI, confidence interval; ePVS, estimated plasma volume status; OR, odds ratio.

### 3.6 ePVS Provides Limited but Statistically Supported Incremental Predictive Value to Traditional Risk Models

**FIGURE 5:**
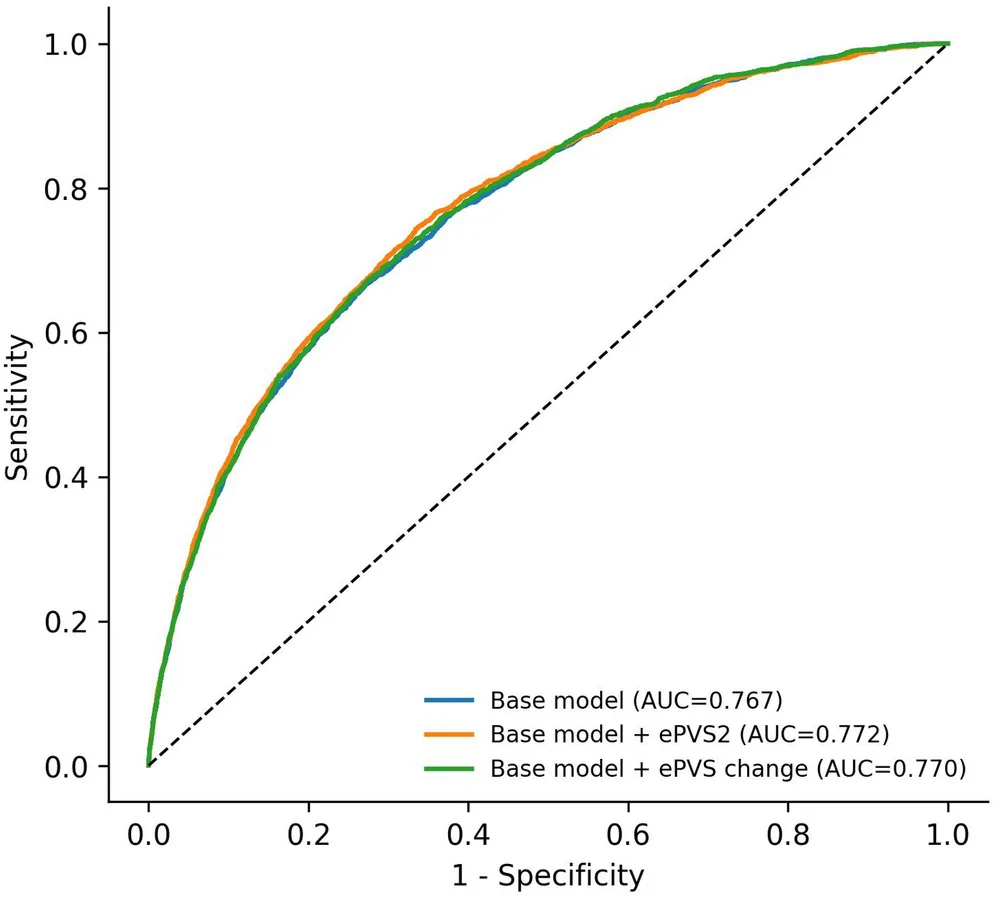
ROC curves of different prediction models. Comparison of the discriminative ability for in‐hospital mortality between the basic model and models adding ePVS_2_ and ePVS change, respectively. The AUC of the base model was 0.767, the base model + ePVS_2_ was 0.772, and the base model + ePVS change was 0.770.

ROC curve analysis indicated that the basic model's discriminatory ability for in‐hospital mortality had an AUC of 0.7666. When ePVS_2_ was added to the basic model, the AUC increased to 0.7721, with a bootstrap AUC delta of 0.0056 (95% CI: 0.0028–0.0085). When ePVS change was incorporated, the AUC was 0.7698, with a bootstrap AUC delta of 0.0033 (95% CI: 0.0016–0.0053) (Figure 5, Table S2). Although the improvement in AUC was limited, the bootstrap confidence intervals for both AUC differences did not cross zero, suggesting small but statistically supported additional discriminatory information beyond traditional clinical variables.

**FIGURE 6:**
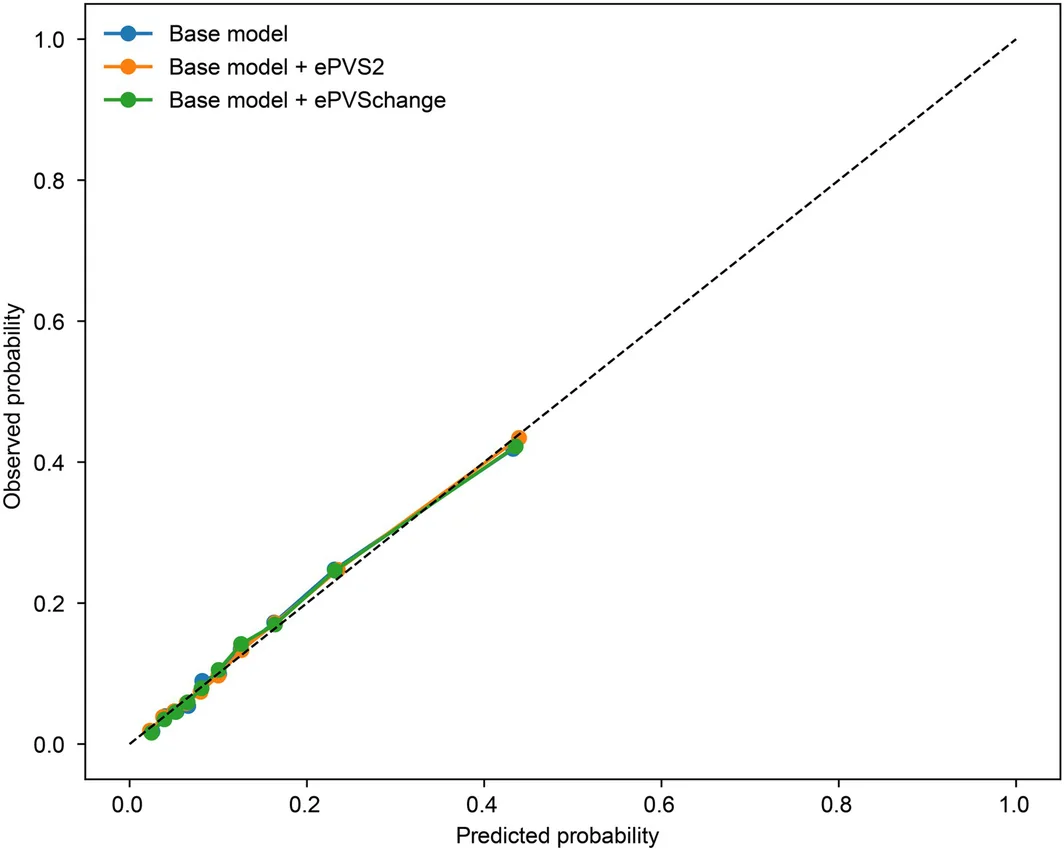
Calibration curves of different prediction models. Comparing the calibration performance of the basic model and the extended models after adding ePVS_2_ and ePVS change. The horizontal axis represents the predicted probability, and the vertical axis represents the actual observed probability. The results show that all models have good calibration, with predicted and observed probabilities concentrated below 0.5 because the overall in‐hospital mortality rate was 13.2% and the predicted‐risk distribution was mostly below this range.

Calibration curves demonstrated good consistency between predicted and observed probabilities for the basic model and the extended models incorporating ePVS_2_ and ePVS change (Figure 6). The predicted and observed probabilities were mainly concentrated below 0.5, which was consistent with the overall in‐hospital mortality rate of 13.2% and the distribution of predicted risk in this cohort. Further NRI and IDI analyses showed that, compared with the basic model, the addition of ePVS_2_ resulted in an NRI of 0.1867 (95% CI: 0.1463–0.2283) and an IDI of 0.0065 (95% CI: 0.0046–0.0086). Meanwhile, the incorporation of ePVS change yielded an NRI of 0.1614 (95% CI: 0.1204–0.2077) and an IDI of 0.0021 (95% CI: 0.0010–0.0035). These findings indicated that ePVS_2_ provided a larger incremental predictive contribution than ePVS change (Figure 5, Figure 6, Table S2).

### 3.7 Higher ePVS_2_ Was Associated With Decreased Short‐Term Survival Rates in Supportive Descriptive Analyses

**FIGURE 7:**
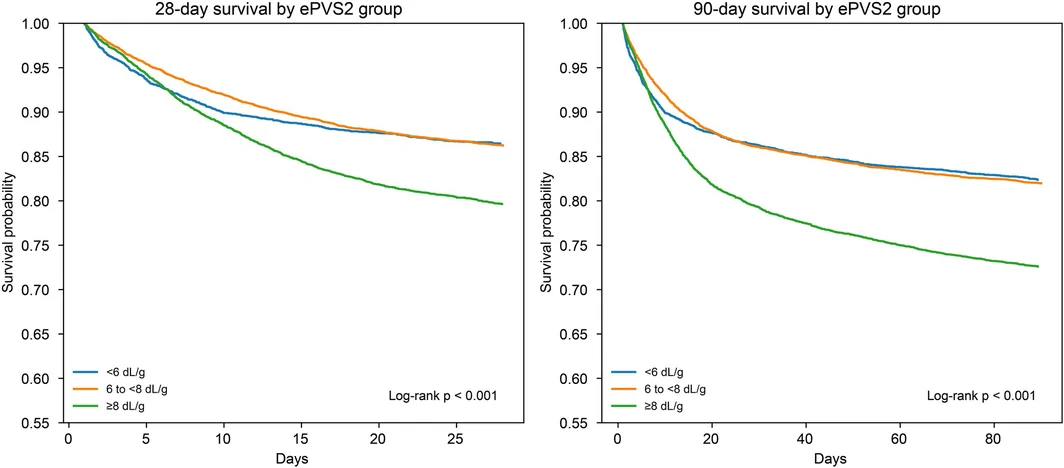
Kaplan–Meier survival curves for different ePVS_2_ groups. The 28‐day and 90‐day survival curves were plotted according to the ePVS_2_ minimum to < 6 dL/g, 6 to < 8 dL/g, and 8 dL/g to maximum groups as supportive descriptive analyses. Follow‐up was indexed from ICU admission; because ePVS_2_ groups were determined using final ICU‐stay values measured after ICU admission, potential immortal‐time‐related bias should be considered when interpreting this figure. There was a significant difference in survival rates among different groups, log‐rank *p* < 0.001.

Kaplan–Meier survival analysis was conducted as a supportive descriptive analysis and revealed significant differences in the 28‐day and 90‐day survival rates among different ePVS_2_ groups (Figure 7). During the 28‐day follow‐up, patients in the ePVS_2_ group from 8 dL/g to the maximum exhibited the lowest survival rate, whereas those in the 6 to < 8 dL/g group had the highest survival rate, with the group from the minimum to < 6 dL/g falling in between. A similar trend was observed during the 90‐day follow‐up. The survival differences among the various groups were statistically significant (log‐rank *p* < 0.001). Because follow‐up was indexed from ICU admission and ePVS_2_ grouping was based on the final ePVS value measured after ICU admission, these curves should be interpreted with consideration of potential immortal‐time‐related bias.

### 3.8 The Risk Association of ePVS_2_ Is Generally Stable Across Different Clinical Subgroups

For ePVS_1_, continuous variable analysis indicated a weak overall effect, with an adjusted OR of 0.98 (95% CI: 0.96–1.00). After stratification by septic shock status, although the direction of the association remained largely consistent, the effect size was limited. In the categorical analysis, the 6–< 8 dL/g group exhibited a lower risk in some subgroups, while the association in the ≥ 8 dL/g group was unstable (Figure S1).

In contrast, the subgroup results for ePVS_2_ were more stable. In the continuous variable analysis, the overall adjusted OR was 1.14 (95% CI: 1.11–1.17). In patients without septic shock and those with septic shock, the adjusted ORs were 1.16 (95% CI: 1.12–1.21) and 1.12 (95% CI: 1.08–1.16), respectively, suggesting that its positive association with in‐hospital mortality existed across different circulatory states. Categorical analysis showed that in patients without septic shock, ePVS_2_ from 8 dL/g to the maximum was associated with a higher risk of mortality. In patients with septic shock, the 6 to < 8 dL/g group had a lower risk than the group from the minimum to < 6 dL/g, and the trend toward increased risk in the group from 8 dL/g to the maximum was attenuated, although the overall direction remained consistent (Figure S2).

Regarding the ePVS change, the overall adjusted OR was 1.04 (95% CI: 1.03–1.05). In patients without septic shock, the association as a continuous variable was stronger [OR 1.07 (95% CI: 1.05–1.09)], whereas the effect was relatively weakened in patients with septic shock [OR 1.02 (95% CI: 1.00–1.03)]. Categorical analysis demonstrated that the range of −4.5 to < 2 exhibited a lower risk in both subgroups, while the range of ≥ 2 showed a more pronounced risk increase, primarily in patients without septic shock (Figure S3). Overall, ePVS_2_ was the most stable indicator across subgroups among the three indices.

### 3.9 Sensitivity Analysis Supports the Robustness of the Main Findings

The results of the sensitivity analysis are presented in Table S3. Taking the impact of a 1 dL/g increase in ePVS_2_ on in‐hospital mortality as an example, the adjusted OR in the primary analysis was 1.138 (95% CI: 1.108–1.169, *p* < 0.001). After excluding extreme values of ePVS_2_ (1st–99th percentile), the OR was 1.104 (95% CI: 1.070–1.138, *p* < 0.001). After excluding patients receiving RRT, the OR was 1.140 (95% CI: 1.109–1.172, *p* < 0.001). After excluding patients with severe anemia (HB1 < 7 or HB2 < 7), the OR was 1.074 (95% CI: 1.041–1.108, *p* < 0.001).

These sensitivity analyses showed that the direction and statistical significance of the association between ePVS_2_ and in‐hospital mortality remained consistent under the predefined restrictive conditions.

## 4 Discussion

Based on the MIMIC‐IV (v2.2) database, this study found that ePVS_2_ and ePVS change were independently associated with in‐hospital mortality, and both showed non‐linear associations with mortality risk. In contrast, ePVS_1_ did not remain independently associated with mortality after adequate adjustment. These findings are consistent with previous studies suggesting the prognostic relevance of ePVS in infection and critical illness [13, 14]. Kim et al. reported that ePVS at ICU admission was associated with in‐hospital mortality in patients with sepsis or septic shock [13], and Gao et al. reported a J‐shaped association between ePVS at ICU admission and 28‐day mortality in septic shock based on MIMIC‐IV data [14]. Compared with these studies, the present study simultaneously evaluated ePVS_1_, ePVS_2_, and ePVS change, thereby focusing on stage‐specific and dynamic plasma‐volume patterns during the ICU course. Because this was an observational single‐center database study, these findings should be interpreted as complementary evidence rather than as proof of causality or as directly generalizable clinical thresholds.

The difference between ePVS_1_ and ePVS_2_ is clinically important. Previous studies have primarily focused on a single ePVS measurement at ICU admission or during early sepsis recognition, which may be strongly influenced by baseline comorbidities, chronic anemia, baseline dehydration, congestive heart failure, and the initial severity of illness [12, 20]. In contrast, the present study showed that early ePVS_1_ lost statistical significance after adjustment for disease severity, organ dysfunction, and treatment factors, whereas ePVS_2_ remained independently associated with in‐hospital mortality. This pattern suggests that late‐ICU ePVS_2_ may better capture the cumulative effects of fluid therapy, disease evolution, organ dysfunction, and treatment response during hospitalization. Therefore, ePVS_2_ should be interpreted as a late dynamic risk marker rather than as a conventional baseline predictor.

The association between higher late‐ICU ePVS_2_, increasing ePVS change, and poor outcomes may also be biologically plausible. A sustained increase in ePVS may reflect hemodilution, persistent positive fluid balance, or failure to achieve effective fluid removal during the stabilization or de‐resuscitation phases of sepsis. Contemporary fluid stewardship concepts emphasize that fluid therapy should progress from resuscitation to stabilization and evacuation, because persistent fluid accumulation can aggravate tissue edema and organ dysfunction [10, 11]. In sepsis, endothelial injury and glycocalyx disruption may further promote capillary leakage, interstitial edema, and impaired microcirculatory perfusion [21]. Thus, a high ePVS_2_ or a marked positive ePVS change may be a clinically accessible signal of ongoing hemodilution and disturbed fluid homeostasis rather than a direct measure of true plasma volume.

The non‐linear findings further indicate that the relationship between ePVS and mortality should not be simplified as a monotonic risk gradient. The relatively low‐risk interval around ePVS_2_ of 6–8 dL/g and ePVS change of −4.5 to 2 should be understood as a statistical risk interval in this cohort, not as a fixed therapeutic target. For clinical interpretation, an ePVS of approximately 8 dL/g may correspond to laboratory combinations such as hemoglobin 8 g/dL and hematocrit 36%, depending on the formula, illustrating that the threshold can be translated into recognizable hemoglobin and hematocrit patterns. The apparent differences between piecewise slope estimates and categorical comparisons should also be interpreted cautiously: the piecewise model estimates local changes along the continuous ePVS scale, whereas categorical models compare average risks across broad intervals. These two parameterizations are complementary but not directly interchangeable.

Regarding predictive performance, adding ePVS_2_ to the baseline model produced only a small increase in AUC, whereas NRI and IDI suggested limited but statistically supported additive discriminatory information. This pattern is consistent with the view that ePVS is more suitable as a supplement to established risk assessment systems rather than as a replacement for mature severity scores such as SOFA and SAPS II. From a clinical perspective, ePVS can be calculated from routine hemoglobin and hematocrit values, which makes it inexpensive and repeatable. In line with the 2021 Surviving Sepsis Campaign emphasis on individualized hemodynamic and fluid management [4], dynamic ePVS monitoring may help support risk stratification and recognition of deterioration, but prospective validation is required before it can be used to guide fluid‐management decisions directly.

The subgroup and sensitivity analyses partly supported the robustness of the main findings. The positive association between ePVS_2_ and in‐hospital mortality was generally consistent in patients with and without septic shock, although shock patients may have more severe circulatory instability, greater treatment intensity, and more complex fluid redistribution, which may attenuate subgroup‐specific estimates. After excluding extreme ePVS_2_ values, patients receiving RRT, and patients with severe anemia, the direction and statistical significance of the association remained stable. Nevertheless, several limitations remain. First, residual confounding and selection bias are unavoidable in a retrospective single‐center database study. Second, ePVS is an estimated indicator derived from hemoglobin and hematocrit and cannot be equated with measured plasma volume. Third, the number of ePVS measurements depended on the availability of paired hemoglobin and hematocrit tests and may have varied according to illness severity. Fourth, ePVS_2_ was based on the final ICU‐stay value, so analyses involving ePVS_2_, especially survival curves, are susceptible to time‐related and immortal‐time‐related bias. Finally, external validation and prospective studies are needed to confirm whether stage‐specific ePVS thresholds can improve clinical decision‐making in sepsis. Future studies with complete timestamped laboratory, vital‐sign, and treatment trajectories should use time‐updated, landmark, or joint longitudinal‐event models to better characterize dynamic ePVS trajectories and reduce time‐related bias.

## Conflicts of Interest

The authors declare no conflicts of interest.

## Supporting information


**Figure S1:** Subgroup analysis of the association between ePVS_1_ and in‐hospital mortality.
**Figure S2:** Subgroup analysis of the relationship between ePVS_2_ and in‐hospital mortality.
**Figure S3:** Subgroup analysis of the relationship between ePVS change and in‐hospital mortality.

**Table S1:** Missing data summary of variables in the cleaned analysis matrix.
**Table S2:** Incremental predictive performance of ePVS indicators: NRI and IDI.
**Table S3:** Sensitivity analyses for the association between ePVS2 and in‐hospital mortality.
**Table S4:** Baseline characteristics associated with ePVS_1_, ePVS_2_, and ePVS change.

## Data Availability

All data generated or analyzed during this study are included in this article and/or its Supporting Information files. Further enquiries can be directed to the corresponding author.
