# Supplementary material for: Non‐Linear Threshold Effect of Dynamic Estimated Plasma Volume Status on Revealing In‐Hospital Mortality Risk in Sepsis
Source: Kaohsiung J Med Sci. 2026 Aug 26:e70278. Online ahead of print. doi: 10.1002/kjm2.70278 (PMC13519500; doi:10.1002/kjm2.70278)
Supplement: Supplementary file 1 [file KJM2-9999-e70278-s001.docx]

**Table S1. Missing Data Summary of Variables in the Cleaned Analysis Matrix.**

| **Variable** | **Missing, n** | **Missing, %** |
| --- | --- | --- |
| PaO2 | 6323 | 36.33 |
| PaCO2 | 6138 | 35.27 |
| pH | 6132 | 35.24 |
| BMI | 5831 | 33.51 |
| Height | 5820 | 33.44 |
| Anion gap (mEq/L) | 2148 | 12.34 |
| Potassium (mEq/L) | 2102 | 12.08 |
| Sodium (mEq/L) | 2089 | 12.0 |
| Bicarbonate (mEq/L) | 1680 | 9.65 |
| Chloride (mEq/L) | 1558 | 8.95 |
| Temperature | 1327 | 7.63 |
| Glucose | 965 | 5.55 |
| Red blood cells (M/μL) | 463 | 2.66 |
| White blood cells (K/μL) | 448 | 2.57 |
| Creatinine (mg/dL) | 419 | 2.41 |
| Urea nitrogen (mg/dL) | 388 | 2.23 |
| Platelet count (K/μL) | 305 | 1.75 |
| Weight | 156 | 0.9 |
| DBP (mmHg) | 117 | 0.67 |
| SBP (mmHg) | 109 | 0.63 |
| Pulse oxygen saturation (%) | 33 | 0.19 |
| Respiratory rate (bpm) | 16 | 0.09 |
| MBP (mmHg) | 2 | 0.01 |
| Heart rate (bpm) | 1 | 0.01 |
| stay_id | 0 | 0.0 |
| Age (years) | 0 | 0.0 |
| Gender | 0 | 0.0 |
| Race | 0 | 0.0 |
| Care_Unit | 0 | 0.0 |
| Hospital length of stay | 0 | 0.0 |
| ICU length of stay | 0 | 0.0 |
| ICU mortality | 0 | 0.0 |
| In-hospital mortality | 0 | 0.0 |
| 28-day status | 0 | 0.0 |
| Time to 28-day outcome | 0 | 0.0 |
| 60-day status | 0 | 0.0 |
| Time to 60-day outcome | 0 | 0.0 |
| 90-day status | 0 | 0.0 |
| Time to 90-day outcome | 0 | 0.0 |
| SOFA | 0 | 0.0 |
| SAPSII | 0 | 0.0 |
| Charlson_Comorbidity_Index | 0 | 0.0 |
| Congestive_Heart_Failure | 0 | 0.0 |
| Myocardial_Infarction | 0 | 0.0 |
| Chronic_Pulmonary_Disease | 0 | 0.0 |
| Renal_Disease | 0 | 0.0 |
| ePVS1_dlperg | 0 | 0.0 |
| ePVS2_dlperg | 0 | 0.0 |
| ePVSmean_dlperg | 0 | 0.0 |
| ePVSchange | 0 | 0.0 |
| HB1 | 0 | 0.0 |
| HCT1 | 0 | 0.0 |
| HB2 | 0 | 0.0 |
| HCT2 | 0 | 0.0 |
| HBmean | 0 | 0.0 |
| HCTmean | 0 | 0.0 |
| Norepinephrine_equivalent | 0 | 0.0 |
| Septic_Shock | 0 | 0.0 |
| AKI_Stage | 0 | 0.0 |
| RRT_24hr | 0 | 0.0 |
| RRT_7day | 0 | 0.0 |
| MV_24hr | 0 | 0.0 |
| MV_48hr | 0 | 0.0 |
| ePVS1_ter | 0 | 0.0 |
| ePVS2_ter | 0 | 0.0 |
| ePVSmean_ter | 0 | 0.0 |
| ePVSchange_ter | 0 | 0.0 |
| Age_65 | 0 | 0.0 |
| AKI | 0 | 0.0 |

Note: Missingness was assessed in the cleaned analysis matrix before modeling. Variable labels were standardized for presentation.

**Table S2. Incremental Predictive Performance of ePVS Indicators: NRI and IDI.**

| **Comparison** | **AUC** | **AUC delta vs base** | **AUC delta 95% CI** | **NRI** | **NRI 95% CI** | **IDI** | **IDI 95% CI** |
| --- | --- | --- | --- | --- | --- | --- | --- |
| Base model | 0.7666 | Reference | -- | -- | -- | -- | -- |
| Base model + ePVS₂ | 0.7721 | 0.0056 | 0.0028–0.0085 | 0.1867 | 0.1463–0.2283 | 0.0065 | 0.0046–0.0086 |
| Base model + ePVS change | 0.7698 | 0.0033 | 0.0016–0.0053 | 0.1614 | 0.1204–0.2077 | 0.0021 | 0.0010–0.0035 |

Note: NRI and IDI compare the incremental predictive value of adding ePVS indicators to the base model including age, sex, Charlson Comorbidity Index, SOFA score, and SAPS II score. CI, confidence interval; NRI, net reclassification improvement; IDI, integrated discrimination improvement.

**Table S3. Sensitivity Analyses for the Association Between ePVS2 and In-Hospital Mortality.**

| **N** | **Events** | **OR** | **95% CI lower** | **95% CI upper** | **P value** | **Exposure** | **Analysis** |
| --- | --- | --- | --- | --- | --- | --- | --- |
| 17403 | 2297 | 1.142 | 1.112 | 1.173 | <0.001 | ePVS2 (per 1 dL/g) | Main analysis |
| 17054 | 2173 | 1.108 | 1.075 | 1.143 | <0.001 | ePVS2 (per 1 dL/g) | Exclude extreme ePVS2 (1st-99th percentile) |
| 17095 | 2153 | 1.141 | 1.11 | 1.173 | <0.001 | ePVS2 (per 1 dL/g) | Exclude RRT patients |
| 16714 | 2107 | 1.078 | 1.045 | 1.112 | <0.001 | ePVS2 (per 1 dL/g) | Exclude severe anemia (HB1<7 or HB2<7) |

Note: Sensitivity analyses were performed using multivariable logistic regression for in-hospital mortality. Models retained the same covariate-adjustment strategy as the main analysis. OR, odds ratio; CI, confidence interval; RRT, renal replacement therapy.

**Table S4. Baseline characteristics associated with ePVS₁, ePVS₂, and ePVS change.**

| **ePVS indicator** | **Baseline characteristic** | **Statistical test** | **Statistic** | **P value** |
| --- | --- | --- | --- | --- |
| ePVS₁ | Age, years | Spearman correlation | 0.083 | <0.001 |
| ePVS₁ | Male sex | Mann-Whitney U | 43501671.5 | <0.001 |
| ePVS₁ | Charlson comorbidity index | Spearman correlation | 0.168 | <0.001 |
| ePVS₁ | SOFA score | Spearman correlation | 0.099 | <0.001 |
| ePVS₁ | SAPS II score | Spearman correlation | 0.087 | <0.001 |
| ePVS₁ | Septic shock | Mann-Whitney U | 35556301.5 | <0.001 |
| ePVS₁ | Acute kidney injury | Mann-Whitney U | 35415913.5 | 0.112 |
| ePVS₁ | Mechanical ventilation | Mann-Whitney U | 39737462.0 | <0.001 |
| ePVS₁ | Renal replacement therapy | Mann-Whitney U | 2208307.5 | <0.001 |
| ePVS₁ | ICU length of stay | Spearman correlation | -0.042 | <0.001 |
| ePVS₂ | Age, years | Spearman correlation | 0.015 | 0.052 |
| ePVS₂ | Male sex | Mann-Whitney U | 42026983.5 | <0.001 |
| ePVS₂ | Charlson comorbidity index | Spearman correlation | 0.113 | <0.001 |
| ePVS₂ | SOFA score | Spearman correlation | 0.145 | <0.001 |
| ePVS₂ | SAPS II score | Spearman correlation | 0.179 | <0.001 |
| ePVS₂ | Septic shock | Mann-Whitney U | 30529356.5 | <0.001 |
| ePVS₂ | Acute kidney injury | Mann-Whitney U | 32206877.0 | <0.001 |
| ePVS₂ | Mechanical ventilation | Mann-Whitney U | 34455930.0 | <0.001 |
| ePVS₂ | Renal replacement therapy | Mann-Whitney U | 1691797.0 | <0.001 |
| ePVS₂ | ICU length of stay | Spearman correlation | 0.197 | <0.001 |
| ePVS change | Age, years | Spearman correlation | -0.026 | <0.001 |
| ePVS change | Male sex | Mann-Whitney U | 36035734.0 | 0.005 |
| ePVS change | Charlson comorbidity index | Spearman correlation | -0.017 | 0.021 |
| ePVS change | SOFA score | Spearman correlation | -0.018 | 0.019 |
| ePVS change | SAPS II score | Spearman correlation | -0.037 | <0.001 |
| ePVS change | Septic shock | Mann-Whitney U | 39667136.5 | <0.001 |
| ePVS change | Acute kidney injury | Mann-Whitney U | 36491242.5 | 0.081 |
| ePVS change | Mechanical ventilation | Mann-Whitney U | 38150127.0 | 0.346 |
| ePVS change | Renal replacement therapy | Mann-Whitney U | 3016246.5 | <0.001 |
| ePVS change | ICU length of stay | Spearman correlation | 0.134 | <0.001 |

Note: Associations between baseline characteristics and each ePVS indicator were examined to support covariate selection and confounding assessment. Spearman correlation was used for continuous baseline variables, and the Mann-Whitney U test was used for binary baseline variables. ePVS, estimated plasma volume status; SOFA, Sequential Organ Failure Assessment; SAPS II, Simplified Acute Physiology Score II; ICU, intensive care unit.
