# Supplementary material for: Non‐Linear Threshold Effect of Dynamic Estimated Plasma Volume Status on Revealing In‐Hospital Mortality Risk in Sepsis
Source: Kaohsiung J Med Sci. 2026 Aug 26:e70278. Online ahead of print. doi: 10.1002/kjm2.70278 (PMC13519500; doi:10.1002/kjm2.70278)
Supplement: Supplementary file 2 [file KJM2-9999-e70278-s002.docx]

**Figure S1. Subgroup analysis of the association between ePVS₁ and in-hospital mortality.**

Note: Forest plots were used to show the association between ePVS₁ as a continuous variable and a categorical variable with in-hospital mortality, stratified by whether complicated by septic shock. Point estimates indicate ORs, and horizontal lines indicate 95% CIs.

**Figure S2. Subgroup analysis of the relationship between ePVS₂ and in-hospital mortality.**

Note: Forest plots were used to display the association of ePVS₂ as continuous and categorical variables with in-hospital mortality, stratified by the presence of septic shock. ePVS₂ maintained a stable association across most subgroups.

**Figure S3. Subgroup analysis of the relationship between ePVS change and in-hospital mortality.**

Note: A forest plot was used to show the association between ePVS change as a continuous and categorical variable and in-hospital mortality, stratified by the presence of septic shock.
